# Application of Variational AutoEncoder (VAE) Model and Image Processing Approaches in Game Design

**DOI:** 10.3390/s23073457

**Published:** 2023-03-25

**Authors:** Hugo Wai Leung Mak, Runze Han, Hoover H. F. Yin

**Affiliations:** 1 Department of Mathematics, The Chinese University of Hong Kong, Shatin, New Territories, Hong Kong, China; 2 Department of Mathematics, The Hong Kong University of Science and Technology, Clear Water Bay, Kowloon, Hong Kong, China; 3 Department of Information Engineering, The Chinese University of Hong Kong, Shatin, New Territories, Hong Kong, China; 4 Department of Electronic and Computer Engineering, The Hong Kong University of Science and Technology, Clear Water Bay, Kowloon, Hong Kong, China

**Keywords:** game design, variational autoencoder (VAE), image and video generation, Bayesian algorithm, loss function, data clustering, data and image analytics, MNIST database, generator and discriminator

## Abstract

In recent decades, the Variational AutoEncoder (VAE) model has shown good potential and capability in image generation and dimensionality reduction. The combination of VAE and various machine learning frameworks has also worked effectively in different daily life applications, however its possible use and effectiveness in modern game design has seldom been explored nor assessed. The use of its feature extractor for data clustering has also been minimally discussed in the literature neither. This study first attempts to explore different mathematical properties of the VAE model, in particular, the theoretical framework of the encoding and decoding processes, the possible achievable lower bound and loss functions of different applications; then applies the established VAE model to generate new game levels based on two well-known game settings; and to validate the effectiveness of its data clustering mechanism with the aid of the Modified National Institute of Standards and Technology (MNIST) database. Respective statistical metrics and assessments are also utilized to evaluate the performance of the proposed VAE model in aforementioned case studies. Based on the statistical and graphical results, several potential deficiencies, for example, difficulties in handling high-dimensional and vast datasets, as well as insufficient clarity of outputs are discussed; then measures of future enhancement, such as tokenization and the combination of VAE and GAN models, are also outlined. Hopefully, this can ultimately maximize the strengths and advantages of VAE for future game design tasks and relevant industrial missions.

## 1. Introduction

In the 21st century, enormous mathematical and data analytic techniques and algorithms have been adopted in designing new video games and frames, for the purposes of enhancing teaching and learning processes in a virtual environment, pushing the innovation and specialty of gameplay mechanics to its furthest extent, and visualizing game scenes in a human-crafted, realistic and dynamic manner [1,2,3]. The concerned subjects include the investigation of 3-dimensional geometric properties of characters within a particular frame [4], the capturing of geometric transformations and motion on a real-time basis [5], and the use of simulation games for analyzing and building up complex systems that could better reflect real world conditions [6]. Today, credited with the increment of computing power and resources, the enhancement of data storage capability, and the massive data volume for simultaneous processing [3], the advancement in machine learning (ML) and artificial intelligence (AI) approaches are taking place and being widely adopted in different practical disciplines, especially those related to image processing and computer vision, as well as the emergence of generative models. This new digital era has also promoted the use of these approaches in handling creative and artistic tasks, for example, a conditional adversarial neural network has been applied for generating city maps from sketch [7]; a Generative Adversarial Network (GAN) model was established to generate images based on the simple sentence description of an object or a specific scenario [8]; the Game Design via Creative Machine Learning (GDCML) mechanism was utilized for setting up an interface with game modules and informing new systems [9]. In view of all these successes, achieving “computational creativity” in the perspective of video game design has now become a hotspot and new focus; while game companies and developers are seeking ways to adopt ML and AI algorithms, so that the overall production cost of a game or related products can be reduced, at the same time, brand new working procedures of the game can also be implemented in the long run. A research report published by Netease has reviewed that the incorporation of ML models into game design could reduce the development costs by millions of Renminbi (RMB) dollars [10].

In the early days of video game development, most games were relatively simple and “monotonous”, and were conducted via the “third-person shooting” mode, with the aid of electronic machines. In 1962, Steve Russell and several student hobbyists at Massachusetts Institute of Technology (MIT) developed the first ever video game in the world, called Spacewar! [11], and this game was published on the Digital Equipment Corporation (DEC) platform at a later time. Within the historical development stage, Spacewar! was considered the first highly influential video game, because it motivated the advancement of computing resources, reviewed the difficulties in transferring programs and graphics between computing platforms at different places [11], and stimulated the development of different game genres. In the early 1970s, the first home video game console called the “Magnavox Odyssey” and the first arcade video game called the “Computer Space and Pong” were respectively established. At the earlier stages, despite the effective integration of technology, creativity and computing resources, there was a lack of uniform standards for classifying game genres in terms of gameplay, however, games can generally be categorized as in [12]. Some key examples include (1) Action Games that emphasize physical challenges, particularly the coordination of hands and eyes; (2) First Person Shooter Games that include the use of guns and weapons for competition and fighting against each other from a first-person perspective; (3) Sports and Racing Games, which simulate the practice of sports or racing originated from real or fantastical environments; (4) Simulation Games that describe a diverse super-category of video games, so that real world activities can be effectively simulated and displayed, for example, flight simulation and farming simulation. With the combination of these genres and capabilities of algorithmic design and data analytics, the importance and popularity of arcades and consoles had diminished, and were gradually replaced by games that are compatible with personal computers, smartphones and mobile devices. Some mainstream game platforms in the 21st century are as shown in Table 1. Nowadays, most games released are not limited to a particular genre, for example, “Need for Speed” is considered a Sports and Racing Game, as well as a Simulation Game [13]; while many games can also be released on multiple platforms, for example, the “Genshin Impact” is compatible with PC, mobile device, and PlayStation simultaneously [14].

Apart from categorizing video games based on their genres and compatible platforms, modern games all consist of three major components, namely (1) the program component; (2) the gameplay component; and (3) the artistic component. Programs form the basis of a video game, which determine the basic structure and logic of the game; gameplays decide how the players and the surrounding environment could interact, via the aspects of designing background settings, battles, balances and stages of the game itself; artistic components lay down what the player can visualize and hear during the gameplay, which can include the design of characters, environmental settings, design of background music and animations, and the ways of interaction [15]. In particular, when designers attempt to produce artistic materials that put spice into the attractiveness of the video game, they may either take reference of real world architectural and parametric settings, or create objects and environments that may not exist in reality. All these have provided possibilities for the utilization and application of image generation techniques within game design processes [16,17].

**Table 1:** Mainstream game platforms and corresponding compatible device in the 21st century.

Game Platform	Company	Device
Personal Computer (PC)	Microsoft	Desktop/laptop computers
Mobile Phone	Apple, Google, Samsung etc.	Smartphones
Xbox [18]	Microsoft	Xbox game console
PlayStation (PS) [19]	Sony	PlayStation 1–5
Switch [20]	Nintendo	Nintendo 3DS/ Nintendo Switch

Recently, designers and scientists have started exploring how ML and combinatorial algorithms could play systematic roles in different levels of game design, for example, data preprocessing, clustering, decoding and encoding, as well as generating attractive and sustainable image outputs in a specific game [21,22,23,24]. In particular, a concept called “game blending” was adopted by Gow and Coreneli to establish a framework that effectively produces new games from multiple existing games [25]; while the Long Short-Term Memory (LSTM) technique has also been applied to blend computer game levels based on Mario and Kid Icarus, then combine with the Variational AutoEncoder (VAE) model to generate more controllable game levels [26,27]. In recent years, Generative Adversarial Network (GAN) models have become popular, and have been incorporated into the framework of generating game levels and images under specific conditions and settings [28,29]. These black-box models allow users to design and generate levels in an automatic manner, thus Schrum et al. [30] utilized such unique features to develop a latent model-based game designing tool; while Torrado et al. [31] investigated the conditional GAN and established a new GAN-based architecture called “Conditional Embedding Self-Attention GAN”, then equipped it with the bootstrapping mechanism for the purpose of generating Role-Playing Games (RPG) levels. On top of this, GANs have been combined with the transfer learning method (e.g., WGAN-GP and BigGAN) to generate new game characters [32], and a 2D game effect sprite generation technique called GESGAN was also established to generate images of prescribed styles and features with a near real-time status [33]. All these have shown the capabilities of ML or deep-learning models in generating game levels within specific set-ups. Nevertheless, it is incredibly hard to obtain a thorough understanding of the internal structure of ML-based models, as well as the statistical properties behind the scene. Therefore, it is of utmost importance to develop and explore the use of a mathematical model that can perform corresponding tasks, i.e., generate new game levels that are applicable in modern game design and for future extension, and at the same time, users can acquire a basic understanding of statistical properties of the model, for example, time complexity, amount of loss during the model training process, and the relationship between time consumption and size of the input dataset. 

In this study, the effectiveness of the Variational AutoEncoder (VAE) model in generating images within game design was first explored and assessed. It is considered a deep generative model that consists of a variational autoencoder, which is equipped with a prior noise distribution. During the model training process, which is usually conducted based on an Expectation-Maximization meta-algorithm, the encoding distribution was “regularized”, so that the resulting latent space sufficed to generate new and meaningful datasets. The detailed mathematical derivation will be discussed in Section 3, and readers can also refer to [34] for more technical details. The VAE model was first proposed by Kingma and Welling [35], and has been widely applied in different disciplines, for example, image generation, data classification and dimensionality reduction [36,37,38]. In particular, Vuyyuru et al. constructed a weather prediction model based on the combination of VAE and Multilayer Perceptrons (MLP) models [39], and Lin et al. attempted to detect the anomaly of office temperature within a prescribed period via LSTM and VAE models [40]. Furthermore, Bao et al. had effectively combined the Convolutional Variational Autoencoder (cVAN) with the GAN model to generate human photos by controlling the gender of required figures [41]. All these case studies have demonstrated the systematic and practical usages of the VAE model, therefore, we expect that with a suitable data processing mechanism, fine-tuning of model parameters, and minimization of the loss function during training, selected game functions or level maps can be generated, as a result provide assistance to game developers in the long run, in terms of auxiliary development, designing new games, and enhancing the speed and time complexity of image generation within specific settings.

Section 2 includes the flowchart of how the VAE model was applied in this study, and the description of datasets used in the three case studies. Then, the mathematical theories and statistical properties of the VAE model are outlined in Section 3, and Section 4 showcases some numerical experiments conducted and their corresponding statistical analyses. Section 5 discusses the deficiencies and limitations of the current study, as well as some potential future research directions; then, a short conclusion is provided in Section 6.

## 2. Flowchart and Data Sources

### 2.1. Overview of This Study

**Figure 1:**
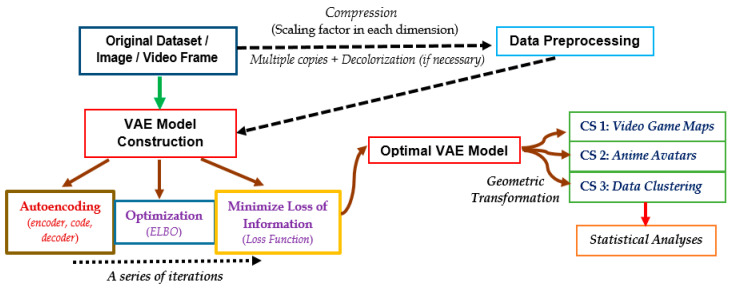
Overall flowchart of this study, from extraction of the original dataset, model construction, and optimization to statistical analyses. CS: Case Study.

Figure 1 shows an overall flowchart of the preprocessing and construction of the VAE model adopted in this study. After raw data or attributes were obtained from games or available databases, they were preprocessed whenever necessary. Upon the application of specific scaling factors within each dimension, these processed datasets could be ingested into a machine, where a sufficient number of datasets was used for model training. In particular, the variational autoencoder within the VAE model was constructed, and the VAE algorithm was iterated such that the optimization of lower bound was achieved under some constraints, which might depend on the set-up of the corresponding game and/or application. Such lower bound was called the “Evidence lower bound (ELBO)”. Further, the loss function during machine learning processes was also minimized, with the aim of estimating the amount of information that has been lost during model training processes. For data clustering applications, an alternative form of the loss function was deemed more appropriate than the aforementioned “loss” during training. After fine-tuning all parameters of such a statistical model, the optimized VAE model was used to obtain some graphical outputs after a series of geometric transformations. In this study, we focus on analyzing the relationship between average loss figures with the number of epochs; the time complexity with the size of input datasets; and the effects of scaling factors, etc. Respective statistical figures are presented either in graphical or tabular formats, as in Section 4.

### 2.2. Data Sources and Description

Within this study, three different datasets have been used for model training and assessing the effectiveness of the developed VAE model. Each of these datasets has its significance, namely, (1) provides good references for game designers; (2) consists of a “humanistic” character equipped with motion; and (3) is practical for image processing and/or data clustering. 

#### 2.2.1. Game Map from Arknights 

Arknights is a tower-defense puzzle game developed by Hypergraph [42]. The game was first published in 2019, and soon became popular and welcomed by many citizens in mainland China. In this study, we attempted to generate new maps based on existing game maps extracted from the official site, which could hopefully provide a useful reference to game designers, especially in updating of the motion and appearance of characters and surrounding spatial features.

**Figure 2:**
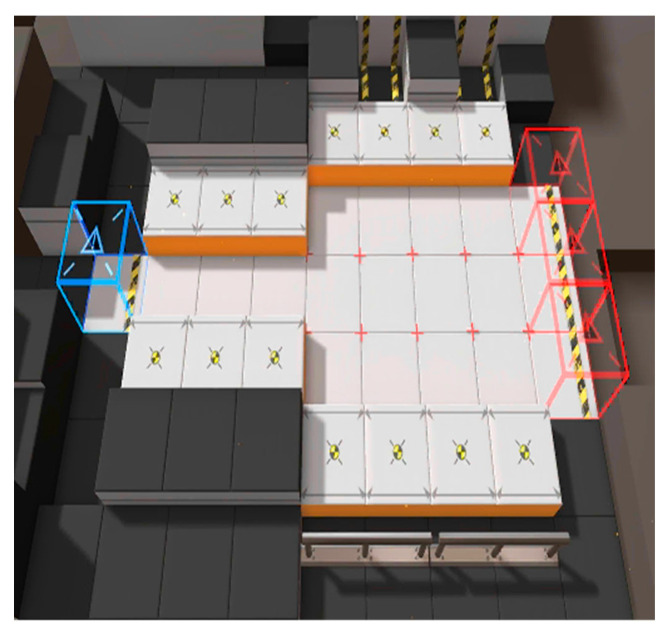
An example of a game map obtained from Arknights via Unity Hub.

A tool called Unity Hub was adopted to dispatch the original game installation package obtained from the official website of Arknights. In total, 180 different game maps were extracted, and Figure 2 shows an example of an original game map image. The size of the original image here is 500 × 500, with 300 pixels per inch (ppi). Detailed documentation of Unity Hub can be found in [43].

#### 2.2.2. Characters from Konachan 

**Figure 3:**
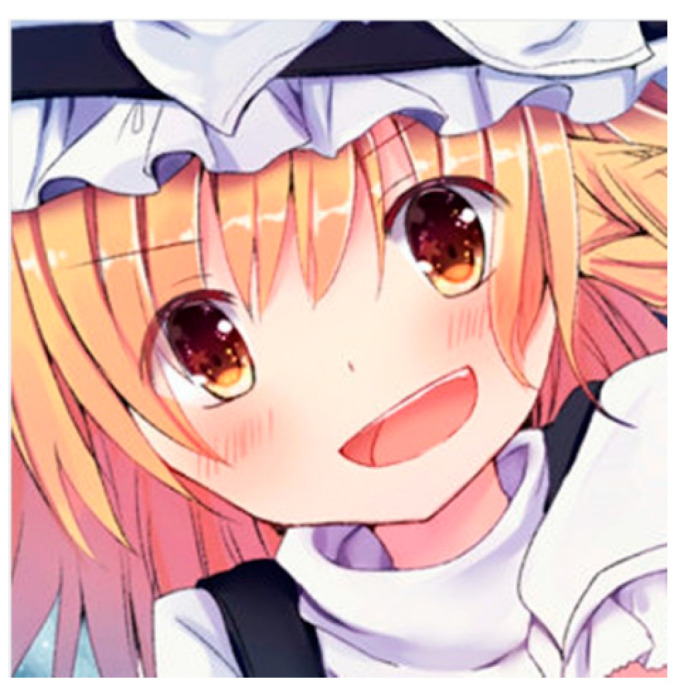
An example of the anime avatar image, from [44].

The second type of dataset(s) adopted in this study was obtained from the Konachan site, which is an image board site that consists of more than 60,000 different anime or manga wallpapers, as of February 2023 [44]. Figure 3 shows an example of an anime avatar extracted from this official website. The size of this image is 512 × 512 digits, with 300 ppi.

#### 2.2.3. Modified National Institute of Standards and Technology (MNIST) Database

The third type of dataset was extracted from the MNIST database, which was created in 1998. The MNIST database contains binary images of handwritten digits and is divided into the training set (Special Database 3) and test set (Special Database 1). The two sets were collected from Census Bureau employees and high school students respectively [45]. This vast database of handwritten digits has been shown useful in pattern recognition and training various image processing systems for classification, with the aid of convolution neural network techniques [46,47]. Original images from MNIST were first being size-normalized, with the corresponding aspect ratio remaining unchanged, so that they could fit into a 20 × 20 pixel box; then, the center of mass of all pixels was computed, so that these processed MNIST images could be positioned at the centre of a “28 × 28 pixel grayscale image” [45]. The database that we adopted in this study consists of 60,000 such grayscale images, each of which consists of 10 digits (from 0 to 9, inclusive), along with a test set that consists of 10,000 images [48]. In this context, the MNIST database was selected to test and validate the effects of clustering, because every data entry has already been pre-labeled with classification labels. 

## 3. Methodologies: Steps of the VAE Model

The important steps and statistical measures of the VAE model are provided in this section, which provide readers with a crucial reference of how the VAE model was constructed; the ideas of data preprocessing; and the important parameters that should be optimized (i.e., maximized or minimized) during machine learning stages. 

### 3.1. Data Preprocessing

First, the raw images were compressed by applying a specific scaling factor, which is defined as the ratio of the length of a side of a desired output image to that of the original image. In this study, a scaling factor of less than 1 was adopted to speed up the machine learning and training processes, at the same time preventing the overflowing of memory. 

Afterwards, the compressed images were decolorized using the optimization approach proposed in [49], with the aim of preserving original color contrasts to the best extent. In principle, the VAE model is applicable for handling RGB images, however, due to the limitations of computer performance, the images obtained from datasets in Section 2 were converted into grayscale styles. Nevertheless, the texture, color contrast and pixel properties were preserved as much as possible, so that the effectiveness of the VAE model could be fairly assessed. In this study, the Intel(R) Xeon(R) CPU E5-2670 v3 (developed by Intel of United States in 2014) with two processors was adopted, and the system was prescribed as a 64-bit operating system, with 128 GB RAM installed. 

As for the Arknights game maps described in Section 2.2.1, since every game map represents only a class label, while a maximum of 180 different images can be obtained from the open data source, therefore, each of these 180 images was copied by 10 times, so that a total of 1800 images were ingested into the VAE model, with most of them being grouped as the ‘training set’, and a small pile of these images was considered the ‘testing set’. Further, the 10 versions of each image possessed different brightness, contrast and gamma correction factors, so that a total of 1800 class labels could be used for conducting statistical analyses.

### 3.2. Autoencoding, Variational AutoEncoder (VAE) and Decoding Processes

**Figure 4:**
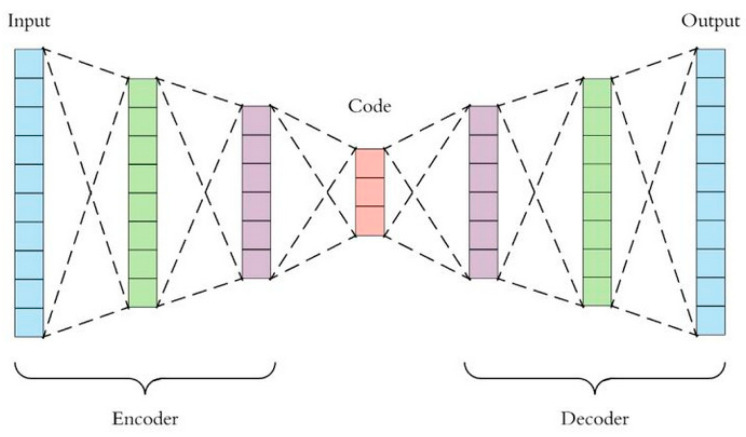
Structure of an autoencoder adopted in the VAE model of this study.

In analyzing large datasets that contain vast number of features within each observation, Principal Component Analysis (PCA) was widely adopted to visualize multi-dimensional information, by reducing the dimension of the original dataset but keeping the maximum amount of information in the output [50]. However, PCA was only applicable in handling linear surfaces, thus the concept of “autoencoding” came in. An autoencoder is capable of handling both linear and non-linear transformations, and is a model that can reduce the dimension of complex datasets via neural network approaches [51]. It adopts backpropagation for learning features at instant time during model training and building stages, thus is more prone to achieve data overfitting when compared with PCA [52]. The structure of an autoencoder is as shown in Figure 4, which includes mainly an encoder to handle input datasets, some codes within the encoding process, and a decoder to produce meaningful outputs.

Denote X as the set of all samples in the original dataset, where $x_{i}$ represents the ith sample. The encoder is a function g(X) that encodes the original dataset to z, i.e., z=g(X), where the dimension of z is significantly less than that of X. Afterwards, the simplified dataset z is passed onto the decoder, which decodes z and outputs $\tilde{X}$. Hence, the decoder is mathematically expressed as $\tilde{X}=f\left(z\right)$. The loss function $l=∥X-\tilde{X}{∥}^{2}$ under arbitrary norm (depending on the type of application) is then used to estimate the closeness between X and $\tilde{X}$. If the magnitude of l is small, the model is considered effective. Here, we may assume that the encoded z will include most valuable information from X, so that z suffices to represent the original dataset even after dimensionality reduction has been applied during the model training process. For example, let $X\in {\mathbb{R}}^{C\times H\times W}$ be an image, where C,H and W are the dimensions that store the information of X. The overall goal is to train an autoencoder that encodes the image into $z\in {\mathbb{R}}^{d}$ (i.e., dimensionality reduction), then apply a decoder that reformulates the image as $\tilde{X}\in {\mathbb{R}}^{C\times H\times W}$ such that the loss function is minimized. In practice, this model will create not only useful attributes of the image, but also unwanted noise components, because the distribution of z, as denoted by p(z), has not been modeled. To complement such deficiency, the Variational AutoEncoder (VAE) was adopted to first model the probabilistic distribution of z, before all useful attributes of X were extracted to form a sampling space of z and passed into the decoder for image recovery.

Suppose z~N(0,I), where **I** represents an identity matrix, which means that z can be regarded as a multi-dimensional random variable that obeys the standard multivariate Gaussian distribution. Denote z and X as random variables, and the corresponding *i*th samples are denoted by $z_{i}$ and $x_{i}$ respectively. With this set-up, the eventual output is generated through a stochastic process of two steps, with z treated as the hidden variable: (1) the prior distribution of X is encoded and sampled to obtain $z_{i}$; then (2) based on the conditional distribution $p\left(X|z_{i}\right)$, a data point or sample $x_{i}$ is achieved. 

As for the decoding process, the samples $z_{i}$ obtained from the N(0,I) distribution were ingested into the decoder, then the parametrized decoder established a mapping that outputs the precise distribution of $z_{i}$ corresponding to X, which is denoted by $p_{\theta }\left(X|z_{i}\right)$. To simplify the statistical complexity, we may assume that X obeys isotropic multivariate Gaussian distribution for any given $z_{i}$, i.e., Equation (1) holds. This means that after $z_{i}$ is ingested into the decoder, the distribution of $X|z_{i}$ can be obtained after fitting $\mu_{i}^{\prime }$ and ${\sigma_{i}^{\prime }}^{2}$.

(1) $$p_{\theta }\left(X|z_{i}\right)=N\left(X|\mu_{i}^{\prime }\left(z_{i};\theta \right),\;\sigma_{i}^{\prime 2}\left(z_{i};\theta \right)*I\right)$$

By taking into account that z~N(0,I), Equation (2) can be obtained, where m represents the hyper-parameter within our VAE model.

(2) $$p_{\theta }\left(X\right)=\int_{z}p_{\theta }(X|z)p\left(z\right)dz\approx \frac{1}{m}\overset{m}{\underset{j=1}{\sum }}p_{\theta }(X|z_{j})$$

Then, the Maximum Likelihood Estimation (MLE) is applied to estimate θ based on the observed or inputted dataset X. The detailed formulation is as shown in Equation (3).

(3) $$\theta^{*}={\mathrm{argmin}}_{\theta }-\sum_{i=1}^{n}\log p_{\theta }\left(x_{i}\right)={\mathrm{argmin}}_{\theta }-\sum_{i=1}^{n}\ln\left(\frac{1}{m}\sum_{j=1}^{m}p_{\theta }\left(X|z_{j}\right)\right)$$

Generally speaking, the dimension of X is very large, while even after the dimensionality reduction process, the dimension of z is not extremely small. Thus, a sufficiently large amount of samples $z_{i}$ have to be considered for achieving an accurate estimate of $p_{\theta }\left(X\right)$. To cope with this, the posterior distribution $p_{\theta }\left(z|x_{i}\right)$ has to be introduced into the encoder. Equation (4) shows how the Bayes’ formula can be applied into computing $p_{\theta }\left(z|x_{i}\right)$. The procedures here are designed and formulated with reference to the ideas proposed in [53].

(4) $$p_{\theta }(z|x_{i})=\frac{p_{\theta }(x_{i}|z)p\left(z\right)}{p_{\theta }\left(x_{i}\right)}=\frac{p_{\theta }(x_{i}|z)p\left(z\right)}{\int_{\hat{z}}p_{\theta }(x_{i}|\hat{z})p\left(\hat{z}\right)d\hat{z}}$$

Next, the AutoEconding Variational Bayesian (AEVB) algorithm is applied to optimize the parametrized encoder and θ. Denote $q_{\phi }\left(z|x_{i}\right)$ as the approximate posterior distribution of the encoder (with parameter ϕ), if $q_{\phi }\left(z|x_{i}\right)\sim p_{\theta }\left(z|x_{i}\right)$, the encoder can be adopted to obtain the probabilistic distribution of $z|x_{i}$ [35]. Since $p_{\theta }\left(X|z\right)$ and p(z) are of multivariate Gaussian distributions, so is $p_{\theta }\left(z|x_{i}\right)$. As a result, it suffices to acquire outputs of μ and $\sigma^{2}$ from the encoder to outline the posterior of the generative model. For any sample $x_{i}$, $q_{\phi }\left(z|x_{i}\right)$ should satisfy the distribution as shown in Equation (5).

(5) $$q_{\phi }(z|x_{i})=N(z|\mu \left(x_{i};\phi \right),\sigma^{2}\left(x_{i};\phi \right)*I)$$

### 3.3. Steps of the VAE Model

Based on the methods reviewed and introduced in Section 3.2, the actual steps of the VAE model in this study are outlined as follows (Steps 1–4):

**Step 1:** The encoder was assigned a data point/sample $x_{i}$, and parameters of $q_{\phi }\left(z|x_{i}\right)$ that the latent variable z obeys were obtained from neural network approaches. Since this posterior distribution is of an isotropic Gaussian distribution, it suffices to find out the parameters $\mu_{i}$ and $\sigma_{i}^{2}$ of the Gaussian distribution that $z|x_{i}$ obeys. As an example, $x_{i}$ here may represent some images of orange cats.

**Step 2:** Based on the parameters $\mu_{i}$ and $\sigma_{i}^{2}$, a sample $z_{i}$ from the distribution was obtained, which is considered a similar type of sample as $x_{i}$. As an example, $z_{i}$ represents all cats that are orange in color. 

**Step 3:** Then, the decoder proceeded to fit the likelihood distribution $p_{\theta }\left(X|z_{i}\right)$, i.e., when $z_{i}$ was ingested into the decoder, the parameters of the distribution that $X|z_{i}$ obeys could be achieved. Since the likelihood would also obey an isotropic Gaussian distribution, we can denote the output parameters as ${\mu_{i}}^{\prime }$ and $\sigma_{i}^{2}\prime$. As an example, $p_{\theta }\left(X|z_{i}\right)$ represents a distribution of images of orange cats.

**Step 4:** After the statistical parameters of the distribution $X|z_{i}$ were acquired, a sequence of data points $\left\{{\tilde{x_{i}}}^{\prime }\right\}$ was obtained via sampling. Nevertheless, most people use ${\mu_{i}}^{\prime }$ as an alternative representation of $\left\{{\tilde{x_{i}}}^{\prime }\right\}$. An example here is to sample a new orange cat image from a particular distribution of orange cats.

In addition, it was also widely recognized that $p_{\theta }\left(X|z_{i}\right)$ is an isotropic multivariate Gaussian distribution with fixed variance, which could be mathematically expressed as in Equation (6), where ${\sigma^{\prime }}^{2}$ is considered a hyper-parameter.

(6) $$p_{\theta }(X|z_{i})=N\left(X|\mu_{i}^{\prime }\left(z_{i};\theta \right),\;{\sigma^{\prime }}^{2}*I\right)$$

**Figure 5:**
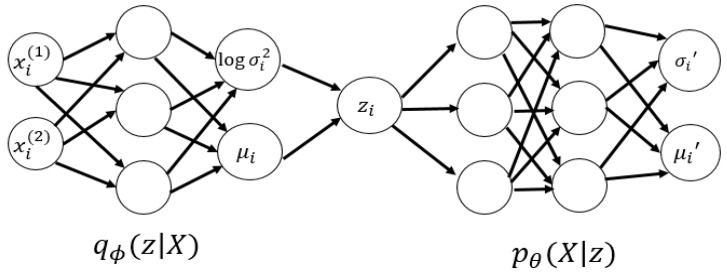
Graphical representation and steps of the VAE model in this study.

The overall graphical structure of the VAE model is as shown in Figure 5.

### 3.4. Evidence Lower Bound (ELBO) of the VAE Model

After fixing the structure of the VAE model for handling datasets in Section 2, an effective loss function for estimating the information loss during model construction process was established. Following the idea of MLE and the application of variational inference, the likelihood function $\ln p_{\theta }\left(X\right)$ can be expressed as in Equation (7), which is bounded below by $l\left(p_{\theta },\;q_{\phi }\right)$. This lower bound is called the “Evidence Lower Bound (ELBO)”.

(7) $$\begin{array}{ll} \ln p_{\theta }\left(X\right) & =\int_{z}q_{\phi }\left(z|X\right)\ln p_{\theta }(X)dz=\int_{z}q_{\phi }(z|X)\ln\frac{p_{\theta }\left(X,z\right)}{p_{\theta }(z|X)}dz\\ & =\int_{z}q_{\phi }\left(z|X\right)\ln\frac{p_{\theta }\left(X,z\right)}{q_{\phi }(z|X)}dz+\int_{z}q_{\phi }(z|X)\ln\frac{q_{\phi }(z|X)}{p_{\theta }(z|X)}dz \end{array}$$

Here, the first integral of the last expression in Equation (7) is denoted as $l\left(p_{\theta },\;q_{\phi }\right)$, while the second integral is called the KL divergence (also known as relative entropy in information theory) and is denoted by $D_{KL}\left(q_{\phi },\;p_{\theta }\right)$. Since KL divergence is always non-negative, $l\left(p_{\theta },\;q_{\phi }\right)$ is considered the lower bound of $\ln p_{\theta }\left(X\right)$. Thus, we have Equation (8) below.

(8) $$\;l\left(p_{\theta },\;q_{\phi }\right)=\ln p_{\theta }\left(X\right)-D_{KL}\left(q_{\phi },\;p_{\theta }\right)$$

That is, to maximize $l\left(p_{\theta },\;q_{\phi }\right)$ is equivalent to maximize $\ln p_{\theta }\left(X\right)$ and to minimize $D_{KL}\left(q_{\phi },\;p_{\theta }\right)$. To minimize $D_{KL}\left(q_{\phi },\;p_{\theta }\right)$, we further assume that the approximate posterior distribution $q_{\phi }(z|x_{i})$ converges to the posterior distribution $p_{\theta }(z|x_{i})$, which is valid because the encoder should only output meaningful distributions for further retrieval and signal recovery in practical implementations. 

Expanding $l\left(p_{\theta },\;q_{\phi }\right)$ as shown in Equation (9), we have the following:

(9) $$\begin{array}{ll} l\left(p_{\theta },\;q_{\phi }\right) & =\int_{z}q_{\phi }\left(z|X\right)\ln\frac{p_{\theta }\left(X,z\right)}{q_{\phi }\left(z|X\right)}dz\\ & =\int_{z}q_{\phi }(z|X)\ln\frac{p\left(z\right)}{q_{\phi }(z|X)}dz+\int_{z}q_{\phi }\left(z|X\right)\ln p_{\theta }(X|z)dz \end{array}$$

Again, the two terms in the last step of Equation (9) have their own physical meanings and implications, where the first integral represents the “latent loss” and is denoted by $-D_{KL}\left(q_{\phi },\;p\right)$; while the second integral is known as the “reconstruction loss” and is denoted by the expectation quantity $E_{q_{\phi }}[\ln\;p_{\theta }(X|z)]$. 

Based on our assumption of the VAE model, $q_{\phi }(z|X)$ and p(z) both follow Gaussian distribution; therefore, the analytical solution of $D_{KL}\left(q_{\phi },\;p\right)$ can be obtained as follows:

(10) $$\begin{array}{ll} D_{KL}\left(N\left(\mu ,\sigma^{2}\right)N\left(0,\;1\right)\right) & =\int_{z}\frac{1}{\sqrt{2\pi \sigma^{2}}}\exp\left(-\frac{{\left(z-\mu \right)}^{2}}{2\sigma^{2}}\right)\ln\frac{\frac{1}{\sqrt{2\pi \sigma^{2}}}\exp\left(-\frac{{\left(z-\mu \right)}^{2}}{2\sigma^{2}}\right)}{\frac{1}{\sqrt{2\pi }}\exp\left(-\frac{z^{2}}{2}\right)}dz\\ & =-\int_{z}\frac{{\left(z-\mu \right)}^{2}}{2\sigma^{2}}N\left(\mu ,\sigma^{2}\right)dz+\int_{z}\frac{z^{2}}{2}N\left(\mu ,\sigma^{2}\right)dz-\int_{z}\ln\sigma N\left(\mu ,\sigma^{2}\right)dz\\ & =-\frac{E\left[{\left(z-\mu \right)}^{2}\right]}{2\sigma^{2}}+\frac{E\left[z^{2}\right]}{2}-\ln\sigma =\frac{1}{2}\left(-1+\sigma^{2}+\mu^{2}-\ln\left(\sigma^{2}\right)\right)\; \end{array}$$

Here, $D_{KL}\left(N\left(\mu ,\sigma^{2}\right)N\left(0,\;1\right)\right)$ represents the relative entropy from N(0, 1) to $N\left(\mu ,\sigma^{2}\right)$ for these two probability distributions defined on the same measurable sample space. 

As for the second term, multiple $z_{i}$’s from $q_{\phi }(z|X)$ are sampled to approximate the term $E_{q_{\phi }}[\ln\;p_{\theta }(X|z)]\approx \frac{1}{m}\sum_{i=1}^{m}\ln p_{\theta }\left(X|z_{i}\right)$, where

$$z_{i}\;\sim \;q_{\phi }\left(z|x_{i}\right)=N\left(z|\mu \left(x_{i};\phi \right),\sigma^{2}\left(x_{i};\phi \right)*I\right)$$

Suppose the dimension of every data point $x_{i}$ is K, we can expand $\ln p_{\theta }\left(X|z_{i}\right)$ as shown in Equation (11) below.

(11) $$\begin{array}{ll} \ln p_{\theta }\left(X|z_{i}\right) & =\ln\frac{\exp\left(-\frac{1}{2}{\left(X-\mu^{\prime }\right)}^{T}\Sigma^{\prime -1}\left(X-\mu^{\prime }\right)\right)}{\sqrt{{\left(2\pi \right)}^{K}\left|\Sigma \prime \right|}}\\ & =-\frac{1}{2}{\left(X-\mu^{\prime }\right)}^{T}\Sigma^{\prime -1}\left(X-\mu^{\prime }\right)-\ln\sqrt{{\left(2\pi \right)}^{K}\left|\;\Sigma \;\prime \right|}\\ & =-\frac{1}{2}\sum_{j=1}^{K}\frac{{\left(X^{\left(j\right)}-\mu^{\left(j\right)}{}^{\prime }\right)}^{2}}{\sigma^{\left(j\right)}\prime }-\ln\sqrt{{\left(2\pi \right)}^{K}\prod_{j=1}^{K}\sigma^{\left(j\right)}\prime } \end{array}$$

### 3.5. General Loss Function of the VAE Model

Based on the parameters introduced in Section 3.4, the loss function L in Equation (12) should be minimized during the machine learning and model training processes:

(12) $$L=-\frac{1}{n}\overset{n}{\underset{i=1}{\sum }}l\left(p_{\theta },\;q_{\phi }\right)=\frac{1}{n}\overset{n}{\underset{i=1}{\sum }}D_{KL}\left(q_{\phi },p_{\theta }\right)-\frac{1}{nm}\overset{n}{\underset{i=1}{\sum }}\overset{m}{\underset{j=1}{\sum }}\ln p_{\theta }(x_{i}|z_{j})$$

In the formula, $z_{j}$’s are actually sampled from $q_{\phi }\left(z|x_{i}\right)$, however, only one such $z_{j}$ is needed empirically, therefore, we simply consider the case of m=1, thus Equation (12) can be simplified as Equation (13).

(13) $$\begin{array}{c} L=\frac{1}{n}\sum_{i=1}^{n}D_{KL}\left(q_{\phi },p_{\theta }\right)-\frac{1}{n}\sum_{i=1}^{n}\ln p_{\theta }(x_{i}|z_{i})\\ \mathrm{where}\;\{\begin{array}{c} \sum_{i=1}^{n}D_{KL}\left(q_{\phi },p_{\theta }\right)=\sum_{i=1}^{n}\sum_{j=1}^{d}\frac{1}{2}\left(-1+\sigma_{i}^{{\left(j\right)}^{2}}+\mu_{i}^{{\left(j\right)}^{2}}-\ln\sigma_{i}^{{\left(j\right)}^{2}}\right)\\ \sum_{i=1}^{n}\ln p_{\theta }(x_{i}|z_{i})=\sum_{i=1}^{n}\left[-\frac{1}{2}\sum_{j=1}^{K}\frac{{\left(x_{i}^{\left(j\right)}-\mu_{i}^{{\left(j\right)}^{\prime }}\right)}^{2}}{\sigma_{i}^{\left(j\right)\prime }}-\ln\sqrt{{\left(2\pi \right)}^{K}\prod_{j=1}^{K}\sigma_{i}^{{\left(j\right)}^{\prime }}}\right] \end{array} \end{array}$$

In our study, by considering that $p_{\theta }\left(X|z_{i}\right)$ is an isotropic multivariate Gaussian distribution with fixed variance, it is reasonable to set $\sigma^{\prime 2}$ as a K-dimensional vector, with all elements being 0.5. With that, the corresponding loss function can be expressed as in Equation (14).

(14) $$L=\frac{1}{n}\overset{n}{\underset{i=1}{\sum }}\overset{d}{\underset{j=1}{\sum }}\frac{1}{2}(-1+\sigma_{i}^{{\left(j\right)}^{2}}+\mu_{i}^{{\left(j\right)}^{2}}-\ln\sigma_{i}^{{\left(j\right)}^{2}})+\frac{1}{n}\overset{n}{\underset{i=1}{\sum }}\left|\left|x_{i}-\mu_{i}^{\prime 2}\right|\right|$$

Here, $x_{i}$ represents the *i*th sample, which acts as the input of the encoder; $\mu_{i}$ and $\sigma_{i}^{2}$ are the outputs of the encoder, which act as the parameters of the distribution of $z|x_{i}$; $z_{i}$ is sampled from $z|x_{i}$ and acts as the input of the decoder; and $\mu_{i}^{\prime }$ is the output of the decoder, which precisely represents the ultimately generated data point $\tilde{x_{i}}$.

### 3.6. Loss Function of the VAE Model in Clustering

As aforementioned, the KL-divergence for $q_{\phi }$ and p is defined as $D_{KL}\left(q_{\phi },p\right)=\int_{z}q_{\phi }(z|X)\ln\frac{p\left(z\right)}{q_{\phi }(z|X)}dz$. Such an expression is only valid when we have the assumptions that q(z) follows Gaussian distribution, and both p(z|X) and q(X|z) follow conditional Gaussian distributions. If all these hold, the loss of the ordinary VAE model can be obtained by a series of substitutions.

Nevertheless, in the case of data clustering, the hidden variables may not always be continuous variables. Thus, we set the latent variable as (z,y), where z is a continuous variable that represents a coding vector, and y is a discrete variable that represents the category. After updating the latent variable, the resulting KL-divergence is as shown in Equation (15), and such an expression is applicable for clustering within the VAE model of this study.

(15) $$D_{KL}\left(q_{\phi }\left(x,z,y\right),p\left(x,z,y\right)\right)=\int_{z}q_{\phi }\left(z,y|X\right)\;\ln\frac{p\left(z,y\right)}{q_{\phi }\left(z,y|X\right)}dz$$

In practice,

(16) $$q_{\phi }\left(z,y|x\right)=q_{\phi }\left(y|z\right)q_{\phi }\left(z|X\right)\;;\;p\left(z,y\right)=p(z|y)p\left(y\right)$$

Based on this, Equation (15) can be re-written as Equation (17), which can essentially obtain the specific loss function of data clustering by following the procedures outlined in preceding sub-sections.

(17) $$D_{KL}\left(q_{\phi }\left(x,z,y\right),p\left(x,z,y\right)\right)=\int_{z}q_{\phi }\left(y|z\right)q_{\phi }\left(z|X\right)\;\ln\frac{p(z|y)p\left(y\right)}{q_{\phi }(y|z)q_{\phi }(z|X)}dz$$

Equation (17) is also applicable for describing both encoding and decoding procedures. First, a data point or dataset X is sampled, which represents an image formed by the original data, then q(z|X) is applied to obtain the encoding characteristic z, followed by the usage of the cluster $q_{\phi }(y|z)$ that classifies the encoded information or attributes. Next, a category y is selected from the distribution p(y), and a random hidden variable z is selected from the distribution p(z|y). Finally, the decoding process can generate new images accordingly. Through these theoretical procedures, images with specific class labels and of minimized loss can be generated in a systematic manner. 

### 3.7. Statistical Metrics and Spatial Assessment

After the VAE model was applied to different case studies, resulting graphical outputs were generated. We first referred to the zoom-in version of these outputs and observed its clarity and features, especially when a game figure or specified character has to be generated. This is considered a type of spatial assessment. As for statistical assessments, we collected and summarized different numerical quantities, including the number of epochs, average loss of information during the image-generation process, the size of input datasets, the scaling factor imposed in the preprocessing stage, and the time consumed for the entire VAE modeling and retrieval process. Then, for attributes that seem to be linearly related, the least-square fitting approach was adopted to find out the best-fit line that describes the relationship between the two concerned quantities. The resulting coefficient of determination (R^2^) is a statistical parameter that ensures the order of accuracy of such a least-squared fit. Such an approach was implemented in statistical analyses of Case Study 1, where the linear fit was applied in investigating the relationship between time consumed with the number of epochs and size of input dataset respectively, as well as the amount of information loss with the choice of scaling factor.

As for numerical quantities that were not linearly related, we connected every two neighboring data points and observed the resulting statistical trends (if any). If possible, the convergence of such a statistical curve will also be our focus, for example, the convergence of average information loss (Case Study 1) and average recognition accuracy (Case Study 3) as the number of epochs increased. These threshold values could be particularly useful for future game designers to determine the optimal settings before model simulations are conducted. 

## 4. Numerical Experiments and Results

### 4.1. Case Study 1: Generation of Video Game Maps

**Figure 6:**
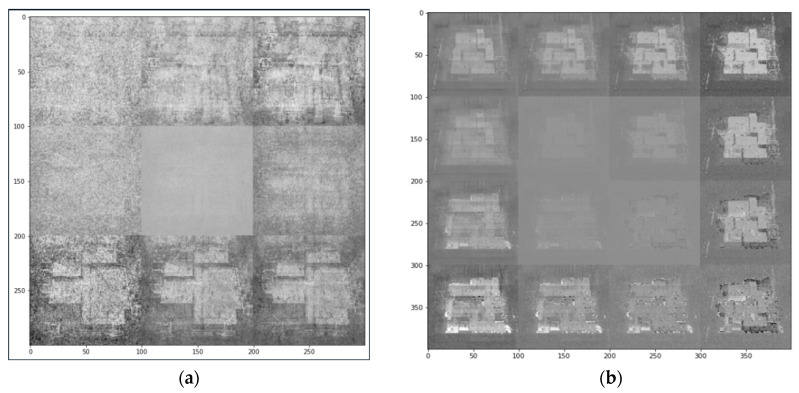
Resulting output game maps obtained after (**a**) the training of the VAE model, and (**b**) the mixing process (based on raw game maps of Arknights obtained from [42]).

**Figure 7:**
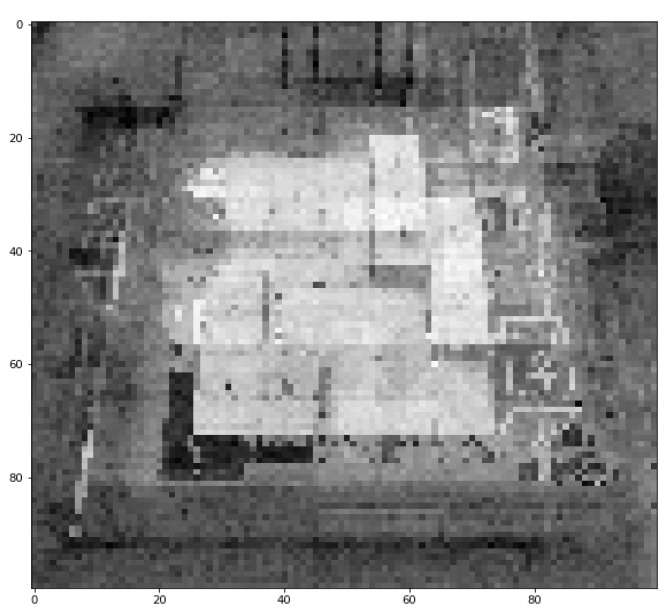
The zoom-in version of an output game map of Arknights after the mixing of several processed images in the VAE model.

The Arknights game maps were downloaded from [42] and were preprocessed as in Section 3.1, where a scaling factor of 0.2 was adopted. Further, the number of epochs within the VAE model was pre-set to be 50, which means that each sample in the training dataset would have 50 times to update its internal model parameters. The number “50” was selected to ensure that the error from the model was sufficiently minimized, but at the same time ensuring the affordability of our computing platform [54]. This number of epochs in testing the effectiveness of a model had been adopted in many studies, such as [55,56], for practical implementations, for example, forecasting power demand and the smart utilization of power grids, and the classification of coronavirus. The VAE-based outputs are as shown in Figure 6 and Figure 7, where Figure 6 shows two outputs obtained after the completion of model training within the VAE architecture and that after the mixing process was conducted; while Figure 7 shows the detailed output game map obtained after several original images were mixed together.

As observed from Figure 6, the images generated by the VAE model could retain the characteristics and features of the original image, and a new image could be effectively generated by mixing several original raw images from [42]. The mixing process was feasible (as shown on Figure 6b). However, when zooming in the output and attempting to visualize the fine details of an image, it was noticed that the sharpness of the output image was rather insufficient, mainly due to the noise components induced during the training process. It is suggested to apply a suitable Laplacian sharpening filter or Sobel gradient for spatial enhancement, so that the edges of figures within the image can become more obvious [57]. Nevertheless, the new output images from the VAE model can still serve as good references for game designers when creating new game levels, or when adding in new characters and spatial features into particular video frames. 

**Figure 8:**
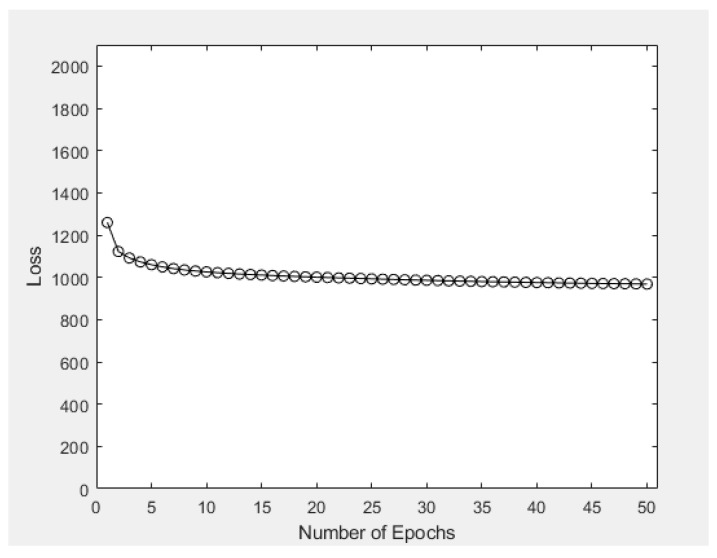
Average loss of the ten image-generation process vs. number of epochs in Case Study 1.

After obtaining the output images, numerical experiments were conducted using the same dataset for exploring the statistical properties of the VAE model. First, Figure 8 shows one of the most well-known learning curves, with the aim of illustrating the relationship between average loss of the ten image-generation process and the number of epochs within the machine learning stage. In general, the average loss of the model decreased as the number of epochs increased, but the rate of decrease of average loss was gradually decreasing with the increase in epochs. Exact statistical figures of average loss within different number of epochs (ranging from 1 to 16, inclusive) are provided in Table 2. The average loss when using 1 epoch was 1259.3, which then decreased to 1122.4 and 1025.6 when 2 and 10 epochs were respectively adopted. Continuing such process, it was observed that at the 21st epoch, the average loss would decrease to 999.0; while after 50 epochs, the resulting average loss would only be 968.24. Overall, both graphical and statistical results have shown that the VAE model is fit to the training dataset extracted from the game map in [42], and 50 epochs is a reasonable number of epochs to be adopted in the VAE model. 

**Figure 9:**
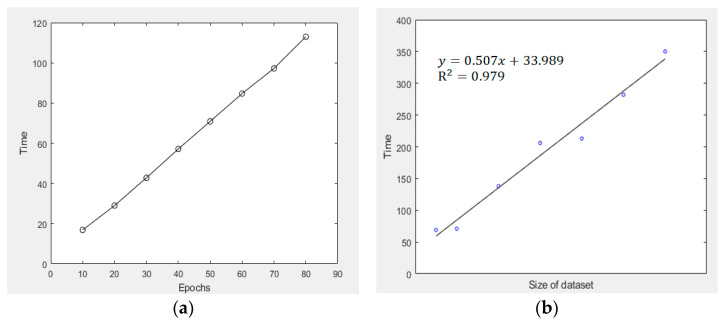
(**a**) Time consumed for VAE model training (in s) versus the number of epochs adopted in the model; (**b**) Time consumed for VAE model training (in s) versus the size of dataset ingested into the model.

Apart from investigating the average loss figures, the amount of time consumed for processing the VAE model is also crucial if one wants to extend the current VAE formulation to handle massive, big datasets in the future. Figure 9a shows the time consumed for training the VAE model against the number of epochs used. The “time” quantity was obtained by taking the average of 10 times of training, with the use of the same dataset and other external parameters for training purposes. The graphical result shows that by fixing all other conditions, the time consumed for VAE model training was linearly related to the number of epochs imposed. For evaluating the time complexity of the VAE model, the number of attributes (i.e., the size of the ingested dataset) was resampled, and the corresponding dataset was ingested into the VAE model, with 50 epochs used during the training process. Then, the time taken for VAE model training was calculated. Figure 9b shows that the R^2^ value of such linear fit between these two quantities is 0.979, which implicates that the time consumed for VAE model training is very likely to be linearly related to data size, thus, the time complexity of the VAE model adopted is of O(*n*).

**Figure 10:**
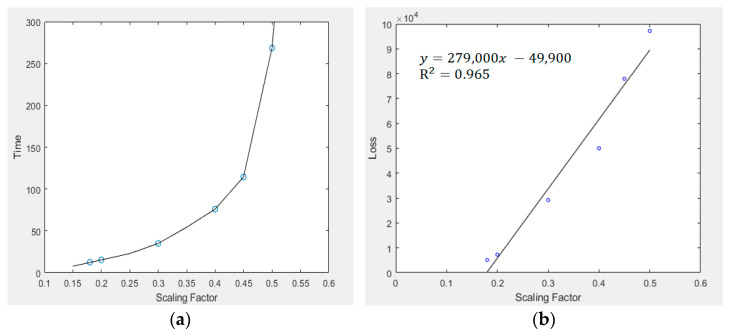
(**a**) Time consumed for ten image-generation process (in s) versus scaling factor imposed in the model; (**b**) Average loss of the ten image-generation process versus scaling factor imposed in the model.

Further, a scaling factor of 0.2 was adopted in this case study. In order to validate the use of such a scaling factor in the VAE formulation, we varied the scaling factor from 0.15 to 0.5 (which was affordable based on the computing platform). For each scaling factor, 10 trial experiments via the application of the VAE model were conducted, and the corresponding time consumed and average loss values were recorded. Figure 10 shows the respective relationship between time consumed for ten image-generation process/average loss figures with respect to the use of different scaling factors. 

As observed, the time consumption was not linearly related to the scaling factor imposed in the VAE model. Instead, the plot shown almost converges to a quadratic or exponential relationship. Nevertheless, as the scaling factor increased from 0.2 to 0.3, the time consumed would exceed double the original time; while when a scaling factor of 0.4 was applied to the VAE model, it took 75 s for processing the VAE model and generating the eventual image. In this case study, there are only limited number of game maps, and the data size of each raw game map is 500×500. Therefore, if this model is extended to handle a large-scale dataset, say those originated from satellite observations [58,59], then it would likely take days or even months for data processing; the same will take place when we extend the VAE algorithm to handle multi-dimensional datasets. Combining this concept with the amount of loss as shown in Figure 10b, a factor of 0.2 was adopted, because a reasonably low average loss was induced by the VAE model, and the computation time for the entire process was not exceptionally long even when dealing with input datasets of larger size. Further, from Figure 10b, the R^2^ value of the resulting linear fit is 0.965; therefore, there is a high possibility that the average loss of the VAE model was linearly related to the scaling factor imposed. 

Despite obtaining all these meaningful conclusions from the correlations between different statistical quantities, we cannot conclude that when a smaller scaling factor is adopted, the model output must be better and of higher clarity. This is because the loss function derived in Section 3.5 only estimates the information loss when comparing the input and output datasets after both encoding and decoding were conducted, but may have ignored the information loss during the preprocessing stage. In actual industrial applications, the information loss of all aspects should be considered, so that an optimal scaling factor can be selected to balance the quality of outputs and model training efficiency.

### 4.2. Case Study 2: Generating Anime Avatars via the VAE Model

**Figure 11:**
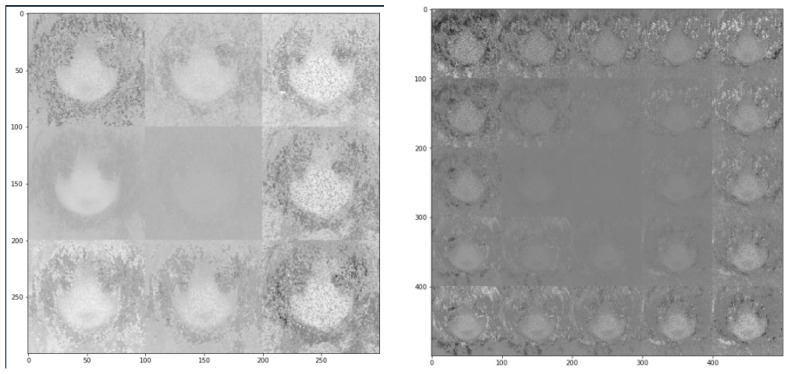
Two sample output images obtained from the VAE model based on the anime-girl dataset (Section 2.2.2).

VAE model is not only useful and applicable in generating a mixture of images or combined game levels from an input dataset, but can also be used to obtain new outputs: If we consider a set of images as the input, after encoding and decoding processes, a totally brand-new image can be created as the eventual output. In this case study, a dataset that consists of 60,000 different anime-girls (an example is shown in Figure 3) was ingested into the VAE model, and the two possible outputs of the model are as shown in Figure 11.

In principle, the creation of new figures or images is not only limited to grayscale representation, but can also be feasible when colored images are desired. The VAE model can generate different new images simply by altering the training dataset, and our purpose within this case study is merely to illustrate the possibility of using the VAE model for generating new datasets or frames. In practice, when colored images are of interests, the computer memory needed will almost be tripled, because the RGB color space requires a 3-dimensional array to record the pixel values (i.e., intensities) of all three different colors.

### 4.3. Case Study 3: Application of VAE Model to Data Clustering

The VAE model is considered both a generative model and a feature extractor, because it consists of an encoder and a decoder (which is treated as a generator), and the distribution of the latent variable can approximately be encoded as a standard normal distribution. Therefore, the effectiveness of the VAE model in performing data clustering was tested, because in principle, the feature extractor could conduct the task without any external supervision. 

**Figure 12:**
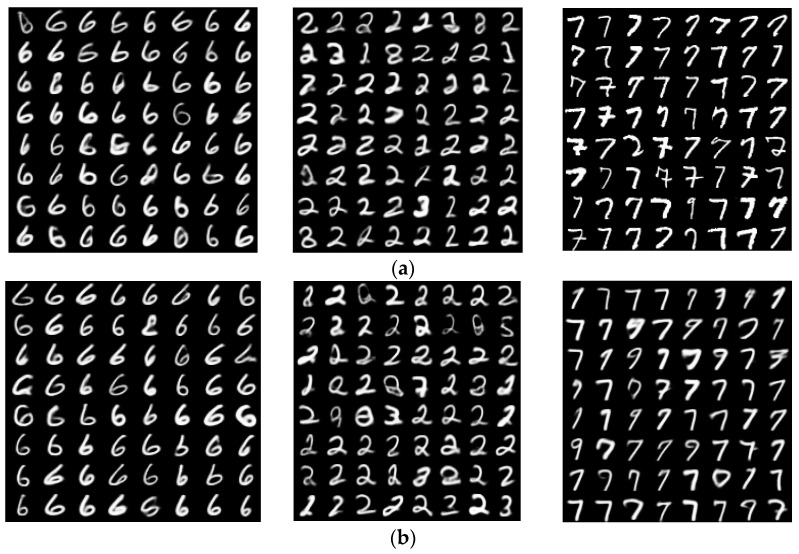
(**a**) Sampled datasets of “6, 2, 7” ingested into the VAE model; (**b**) Output datasets obtained from the VAE model.

In this study, the MINST dataset of handwritten numbers (described in Section 2.2.3) was used to illustrate the applicability of the VAE model in data clustering. Figure 12a shows the sampled numbers “6, 2, 7”, while Figure 12b showcases the corresponding numbers generated by the VAE model after data clustering was applied to all numbers shown in Figure 12a. Obviously, the VAE model had reasonably good performance in terms of data clustering, and was capable of classifying different data types without any supervision, then generating appropriate images that correspond to the clustered datasets. 

Within this case study, 50 epochs was adopted, and the averaged recognition accuracy based on conducting 10 similar experiments via the VAE model was 85.4%. The corresponding training accuracy was around 83.7%. Table 3 shows the average accuracy of data clustering when different numbers of epochs were applied. 

**Figure 13:**
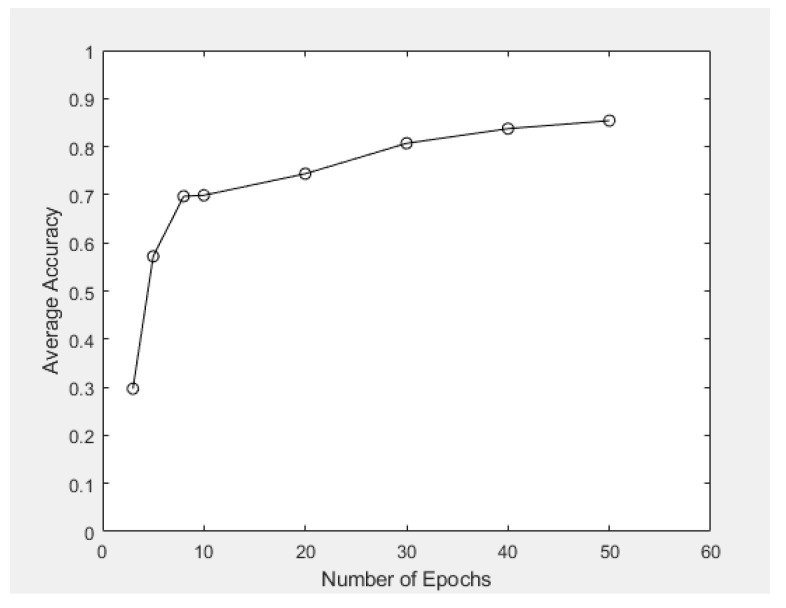
Average recognition accuracy versus the number of epochs used in the model.

When the number of epochs increased from 3 to 5, significant improvement with respect to the performance of data clustering was achieved, where the average accuracy increased abruptly from 29.7% to 57.2%. Figure 13 displays the associated graphical relationship between these two quantities, which verifies that (1) the average recognition accuracy was enhanced as the number of epochs increased; and (2) as the number of epochs increased, the increment in average accuracy decreased, and the average accuracy converged to a threshold bounded above by 0.9 in this case study. 

In actual gameplay, game designers can make use of the VAE model to recognize specific patterns of images or video frames, for example, the automatic recognition of sketches from game players. On top, the VAE model is also capable of performing data augmentation, which is particularly useful for puzzle games, where players are required to “draw” an object or “write down” an answer. Once the pattern resembles the model answer, the game will treat the player as “correct” and give out an award, or upgrade the player to more advanced stages of the game. The VAE model can be fully utilized to serve related purposes, for example, pattern recognition and the clustering of objects or datasets. 

### 4.4. Insights from Results of Case Studies & Practical Implementation

Figure 8 and Table 2 in Section 4.1 associate the average loss figure induced with the number of epochs. The data were obtained based on a scaling factor of 0.2, and the number of epochs adopted in the VAE model (for training and prediction, etc.) was 50. It was observed that when formulating new game levels or creating new frames, the average loss figure would have become steady and eventually converged to a limiting value (around 950). This indicates that the use of these parameters in VAE modeling is generally acceptable. Nevertheless, as shown in Figure 9a, the time consumed for VAE model training was linearly related to the number of epochs. This means that a larger number of epochs is feasible in real-life implementation if one can wait for a longer period of time. In terms of data clustering in Section 4.3, the average accuracy had a sharp increase when the number of epochs increased from 3 to 30 (from around 0.3 to 0.8), but then, the increasing trend became steadier when the number of epochs increased from 30 to 50, and the average accuracy eventually converged to around 0.85 (as shown in Figure 13). This indicates that 50 epochs or above would be practical enough for image generation, creation of new game levels, and even data clustering.

As for the choice of scaling factor, as shown in Figure 10a, when it ranged from 0.15 to around 0.4, the time taken for new image generation was increasing at an almost linear trend. However, when the scaling factor exceeded 0.4, an excessive increment of time would have taken place. Further, from Figure 10b, when the scaling factor was 0.18 or 0.2, the average loss of information or input attributes would be similar, however, when the scaling factor increased to 0.3, the average loss was tripled. Such an experimental testing could explain why the scaling factor of 0.2 should be adopted when designing new game levels, and such a factor must not exceed 0.4 in all practical implementations when the VAE model is going to be involved in model development or training processes. Regarding the model explainability of VAE, since all images or video frames that we considered were obtained from real observations of corresponding games, corresponding sub-centroids could be summarized and treated as actual training images within the model, then these data points or features could also be of practical usage during feature classification. This “ad-hoc explainability” concept was validated in the recently established deep nearest centroids (DNCs) model [60], where human-understandable explanations could be effectively derived. This was actually quite similar in our VAE model, where the sub-centroids of each image pixel could also be computed and identified.

## 5. Discussions and Limitations

### 5.1. Deficiencies of a Low-Dimensional Manifold & Tokenization 

Although the applicability of the VAE model in modern game design, pattern recognition, and data clustering was clearly illustrated in this study, there is some room for improvements based on the graphical results obtained from some of our case studies. In particular, in the case study of Arknights, when zooming Figure 7 into details, the image quality at specific pixels or regions could be dissatisfactory. This is because the input image consisted of some discrete pixels or point clouds in a high-dimensional space, and VAE attempted to first compress them into a low-dimensional continuous space (which was denoted as a “latent space”), then restore the original space via the decoding process. It was observed that VAE could work very well when the input dataset is actually a low-dimensional manifold embedded in a higher-dimensional space [61], however, some graphics, such as those in Arknights, are obviously not a low-dimensional manifold itself in nature. This has led to some potential errors within the VAE-based retrieval process. Further, some features of images, such as texture, are relatively hard to be described with a low data volume, but texture can indeed play an important role in computer vision applications, for example, surface detection and medical imaging [62]. Therefore, to enhance the quality of outputs from the VAE model in these industrial applications, other deep-learning networks and transform-based methods can be adopted to distinguish these features at an early stage, either via the use of a smooth function for transformation, or extracting the concerned features in another space, with the aid of wavelet transforms [63], ridgelet transform [64], or a Gabor filter [65]. Then, the corresponding attribute(s) can be combined with the latent space vector in VAE to produce better numerical results, and the information lost during encoding and decoding processes of VAE can also be minimized. For label distribution construction, the spherical Fibonacci lattice algorithm proposed by González [66] can be used for point sampling and obtaining a distribution that possesses unbiased expectation. Afterwards, the loss function introduced in [67] can be introduced into the modeling framework, with an attempt to understand the corresponding parameters of each input sample.

Further, in order to filter off the invalid attributes and simplify the useful information from the original input dataset, instead of generating low-dimensional images in all scenarios, researchers have proposed dividing a particular game map into the combination of different map elements or components, then replacing these components by some tokens. This process is known as “tokenization”, as described in [68]. Much simplified new images that retain all useful attributes could be constructed, then the prescribed machine learning or VAE model can be used to train these images and produce combined outputs. In the future, this technique can be incorporated into the existing VAE model for enhancing image resolution and producing images with better quality, especially for images similar to our Case Study 1. 

### 5.2. Image Compression, Clarity of Outputs & Model Training

In the VAE model, a scaling factor has to be applied to raw datasets during the data preprocessing stage. If one excessively compresses the original image, much useful information will be lost, and fine details cannot be effectively kept during the model training stage, which could result in outputs of insufficient clarity. On the other hand, if the compression was not conducted, huge computing resources would be occupied especially when we are handling large-scale datasets, for example, the database from ImageNet [69] or remotely sensed imageries for object detection or environmental monitoring [70,71]. In most desktops, the memory is only of 4–48 G [72]; therefore, memory overflow will easily take place, thus limiting the overall efficiency and reliability of a model. The time consumed for model training and image retrieval will be excessively long as well. On top of that, in terms of model training, it will take many rounds of data analytic experiments in order to optimize the hyper-parameters of the VAE model, and as a result, increase the overall time consumed. Therefore, it is of utmost importance to strike a balance between the quality of outputs and the time consumed for generating the outputs via modeling approaches.

For the purposes of game design and creating new game levels, in order to alleviate the problem of insufficient clarity caused by the VAE model, and to avoid the occurrence of “mode collapse” (i.e., only one or several image types will be generated) that often takes place in traditional GAN models, the combination of VAE and GAN models can be adopted. The VAE model only consists of one generator, while the GAN model consists of both a generator and a discriminator. These two “machines” oppose each other, where the generator is continuously attempting to generate images and frames that can fool the discriminator; as a result, the probability for a discriminator to make mistakes will increase; while the discriminator tries its best to distinguish between real and useful data from fake ones via appropriate neural network mechanisms [73]. As a result, better outputs can be generated after a series of to and fro opposed checking. For enhancing the clarity of images in Case Studies 1 and 2 of this paper, we propose adopting the VAE model as the generator and simultaneously develop a discriminator to supervise the VAE model, i.e., a combined version of VAE and GAN models is to be established. As a result, the generator of the VAE-GAN model will consist of the statistical or probabilistic distribution of the original input dataset, and at the same time, it can effectively reduce the training time throughout the entire process and minimize the chance of the model suffering from “mode collapse”. 

## 6. Conclusions

In this study, we illustrated the possibility and statistical feasibility of using the combination of a VAE model and machine learning strategies for modern game design, with the aid of three case studies arising from different natural scenarios and applications. The mathematical principles and assumptions of the VAE model, as well as its Evidence Lower Bound (ELBO), loss function during model construction, and loss function in data clustering, were first explored and derived. Then, the VAE model was applied to generate new game maps based on existing maps obtained from Arknights, create and retrieve anime avatars, and cluster a group of MNIST datasets that consist of numerals. The output images and datasets could retain and re-combine information from the inputs to a certain extent, however, in the case study of Arknights (Case Study 1), there was room for improvements due to the lack of clarity in terms of the output image, which could essentially represent a new game level in practice.

Some statistical features of the model and the relationship between different parameters were also reviewed from these three case studies, for example, there was a high possibility that the time complexity of this VAE model is O(*n*); the loss of the VAE model decreased as the number of epochs applied increased, but the rate of change of such loss was also declining in general; and the time consumed for performing the VAE model was positively and linearly related to the number of epochs. For preventing memory overflow and saving computing resources, an appropriate scaling factor had been applied to each input dataset or image at the preprocessing stage. It was found that the time consumed increased as the scaling factor increased, and it was quite clear that the loss derived from the loss function was positively and linearly related to this scaling factor. 

Despite showing some technical deficiencies in generating new game levels (as reviewed in Case Study 1), the VAE model has shown its capability in data clustering. Further, for image attributes (or data points) with obviously different characteristics or spatial features, the VAE model can also successfully distinguish one class from another via the model training process, then generate images of a specific class. On average, the recognition accuracy under 50 epochs is 85.4%, which is considered satisfactory.

Generally speaking, the VAE model is most effective in generating images with a specific graphical pattern, or handling and producing images of low resolution requirements, for example, clouds, grass and distant views in our nature. It is particularly promising in terms of clustering and creating new characters within a game.

In view of the technical shortcoming of the current VAE model, we have learnt that the future enhancement should focus on increasing the resolution of images generated, for example, via the combination of the VAE model with other machine learning mechanisms, such as GAN and LSTM, ensuring sufficiency with regard to the amount of information in the model training set, so that all output images will contain more useful information and attributes, but at the same time consist of the least amount of noise components. This may be possible by tracing back to the techniques adopted in data preprocessing stages. This study has opened a new window for utilizing the strengths of VAE for future game design missions within the industry, at the same time identifying some potential weaknesses of VAE and proposing potential ways to remedy these deficiencies in the foreseeable future. 

## Figures and Tables

**Table 2:** Statistical figures of average loss at different number of epochs within the VAE model.

Number of Epochs	Average Loss	Decrease in Average Loss with 1 More Epoch
1	1259.3	Not applicable
2	1122.4	136.9
3	1091.5	30.9
4	1072.9	18.6
5	1059.7	13.2
6	1049.2	10.5
7	1041.1	6.9
8	1034.8	6.3
9	1029.8	5.0
10	1025.6	4.2
11	1021.9	3.7
12	1018.7	3.2
13	1015.5	3.2
14	1013.1	2.4
15	1010.7	2.4
16	1008.4	2.3

**Table 3:** Relationship between average accuracy (%) in data clustering and the number of epochs used in the VAE model.

Number of Epochs	Average Accuracy
3	29.7
5	57.2
8	69.7
10	69.9
20	74.3
30	80.7
40	83.7
50	85.4

## Data Availability

The data presented in this study are available on request from the corresponding authors.
